# Enhancing spatiotemporal focusing of light deep inside scattering media with Time-Gated Reflection Matrix

**DOI:** 10.1038/s41377-022-00858-w

**Published:** 2022-06-01

**Authors:** Zhipeng Yu, Huanhao Li, Tianting Zhong, Puxiang Lai

**Affiliations:** 1 Department of Biomedical Engineering, The Hong Kong Polytechnic University, Hong Kong SAR, China; 2 Shenzhen Research Institute, The Hong Kong Polytechnic University, Shenzhen, China; 3 Photonics Research Institute, The Hong Kong Polytechnic University, Hong Kong SAR, China

**Keywords:** Optical techniques, Optical physics

## Abstract

Time-gated reflection matrix (RM) has been successfully used for optical imaging deep inside scattering media. Recently, this method was extended to enhance the spatiotemporal focusing of light ultra-deep inside scattering media. This is achieved by calibrating the decomposition of the RM with the Tikhonov regularization parameter to convert multiply scattered photons that share the same time of flight with the singly scattered photons into singly scattered photons. Such a capability suggests a reshaping to the interaction mechanism between light and scattering media, which may benefit or inspire wide optical applications that desire enhanced spatiotemporal focusing of light at depths inside scattering media.

Light is playing an increasingly important role in biomedicine from imaging, sensing, therapy, stimulation, to manipulation. The applications, however, have seen limitations at depths in biological tissue and tissue-like scattering media, where photons experience multiple scattering events due to the spatial inhomogeneities of refractive index. It is possible to exploit multiply scattered (MS) photons for imaging in some techniques, such as diffuse optical tomography^1^, ultrasound-modulated optical tomography^2^, and photoacoustic tomography^3^, albeit with compromised resolution. To achieve a high resolution, singly scattered (SS) photons (also known as ballistic photons) that only probe the in situ target need to be screened out from the predominant MS photons^4^. Endeavors to this purpose can be divided into two categories. In the first category, SS photons are spatially filtered out from MS photons, and the representative technology is confocal microscopy where a small pinhole aperture only allows fluorescence emission from the desired focal plane to pass through^5^. In the second category, SS photons are temporally filtered out from MS photons, as demonstrated in optical coherence tomography (OCT) based on time-gating technique^6^. For both categories, the imaging depth are still restricted to ~1 mm beneath tissue surface because the number of SS photons decreases exponentially with optical thickness^7^.

Inspired by the transmission matrix approach^8^ and random matrix model in ultrasound imaging through strongly scattering media^9^, a time-gated reflection matrix (RM)-based method called “Smart OCT” was proposed to enhance the imaging depth^10,11^. In this implementation, a singular value decomposition of the RM was used to screen out most of the MS photons. Despite of that, MS photons with the same time of flight as the SS photons were still dominant for targets located deeper than a few scattering mean free paths (SMFP). The common wisdom is that the residual MS photons have impeded the imaging quality and hence in order to yield high resolution, they need to be removed or suppressed through methods such as iteration^12^ or a spatial input–output correlation^11^. Most recently, in *Light: Science & Applications*, Cao et al. suggest that the part of MS photons that share the same time of flight as the SS photons can be utilized and converted into SS photons to enhance in situ optical energy delivery spatially and temporally^13^.

In this work, the main goal is to retrieve singular values of the part of MS photons with the same time of flight as the SS photons. First, a coherent gating is created inside the scattering medium using an ultra-short pulse beam. The back-reflected photons from the scattering medium can be divided into three types: SS photons (ξ_SS_), MS2 photons (ξ_MS2_) sharing the same time of flight as the SS photons, and other remaining MS photons (ξ_MS1_). After the construction of RM, a singular value decomposition (also termed as “time reversal operator”) is applied to the RM (R): $R = U\Sigma V^{\rm T}$, where *T* represents transpose, Σ is a diagonal matrix containing the real positive singular values in a descending order $\sigma _1 \,>\, \sigma _2 \,>\, \cdots \,>\, \sigma _N$ (*N* is the number of the singular values in Σ), *U* and *V* are two unitary matrices whose columns correspond to the input and output mode, respectively. Singular values in the diagonal matrix corresponding to the SS, MS2, and MS1 photons are also in a descending order. Practically, Σ is not actually a standard diagonal matrix and it cannot be used to filter out the MS2 photons. Thus, matrix$R^{\dagger} R = USV^T$(${\dagger}$ represents conjugate transpose) that has a more standard diagonal matrix will be taken into consideration in the inversion process. As a result, *S* is a diagonal matrix containing the square of the singular values ($\sigma _i^2$) of the diagonal matrix Σ. Note that however, this operator is very labile in ultra-deep position due to the noise and there are a lot of non-zero elements at adjacent positions of diagonal line of the diagonal matrix. To reduce the influence of noise, the Tikhonov regularization parameter^14^ is introduced to create a calibration matrix to optimize the reversal results in the inversion process. The calibrated matrix is $C = UFSV^T$, where *F* is a diagonal matrix with diagonal elements $\alpha _i = \sigma _i^2{{{\mathrm{/}}}}\left( {\sigma _i^2 + \lambda ^2} \right)$ (*λ* is a variance ranging from 10^−8^ to 10^8^ for different penetration depths). During the selection of *λ*, there is a tradeoff between the retrieval number of eigenstates and the retrieval accuracy rate of each eigenstate. In the optimization process, the target is to make the output field from calibrated matrix close to the measured output field. After successfully retrieving singular values of the SS and MS2 photons, the desired wavefront can be acquired, and the corresponding phase pattern will be loaded on the spatial light modulator. At last, the optical energy delivery can be enhanced by a magnitude at an ultra-deep (~14.4 SMFP) position.

As demonstrated in this study, the optical energy delivery can be enhanced by shaping some part of the MS photons into SS photons, it can be potentially used to increase the signal-to-noise ratio (SNR) or the imaging depth of “Smart OCT”. Fundamentally, optical scattering arises from the interaction between photons and matter. Therefore, shaping MS photons into SS photons suggests that the light-matter interaction for the particular photons is changed and furtherly, the underlying physical mechanism can be reshaped from the conventional realm. This method may also benefit or inspire other optical applications that desire enhanced spatiotemporal focusing of light at depths inside scattering media, such as selective optogenetics^15^ and laser microsurgery^16^, etc.
